# The Immediate Effect of a Single Treatment of Neuromuscular Electrical Stimulation with the StimaWELL 120MTRS System on Multifidus Stiffness in Patients with Chronic Low Back Pain

**DOI:** 10.3390/diagnostics14222594

**Published:** 2024-11-19

**Authors:** Daniel Wolfe, Geoffrey Dover, Mathieu Boily, Maryse Fortin

**Affiliations:** 1 Department of Health, Kinesiology, and Applied Physiology, Concordia University, Montreal, QC H4B 1R6, Canada; 2 School of Health, Concordia University, Montreal, QC H4B 1R6, Canada; 3 McGill University Health Centre, Montreal, QC H4A 3J1, Canada; 4 Centre de Réadaptation Constance-Lethbridge du CIUSSS du Centre-Ouest de l’Île-de-Montreal, Montreal, QC H4B 1T3, Canada

**Keywords:** chronic low back pain, shear-wave elastography, lumbar multifidus

## Abstract

Background/Objectives: Individuals with chronic low back pain (CLBP) have altered lumbar multifidus stiffness properties compared to healthy controls. Although neuromuscular electrical stimulation (NMES) application to the multifidus might affect stiffness, this has never been investigated. The aims of this study were to examine the effect of a single NMES treatment on multifidus stiffness and pain intensity in CLBP patients. Methods: 30 participants (13 male, 17 female) were randomized to one of two intervention (‘phasic’ and ‘combined’) protocols with the StimaWELL 120MTRS system. Multifidus stiffness at L4 and L5 was measured via shear-wave elastography (SWE) at rest and in standing prior to, and 15 min after, a 20 min NMES treatment. Pain intensity was measured pre- and post-treatment with the numerical pain rating scale (NPRS). Results: There were significant increases in resting shear modulus at right L4 (*p* = 0.001) and bilaterally at L5 (*p* = 0.017; *p* = 0.020) in the ‘combined’ intervention group, and a significant between-group difference at right L4 (*p* < 0.001). There were significant decreases in standing shear modulus at right L4 (*p* = 0.015) and left L5 (*p* = 0.036) in the ‘combined’ intervention group, and a significant between-group difference at left L5 (*p* = 0.016). Both groups experienced significant decreases in pain intensity (MD combined group = 1.12, 95% CI [0.34, 1.90], *p* = 0.011) (MD phasic group = 1.42, 95% CI [0.68, 2.16], *p* = 0.001). Conclusions: There were multiple significant changes in multifidus stiffness in the combined group, but not in the phasic group. Both groups experienced significant decreases in low back pain intensity.

## 1. Introduction

Chronic low back pain (CLBP) is the leading cause of disability worldwide [1,2]. CLBP is a multi-factorial condition, and its biological markers include intervertebral disc degeneration [3], vertebral endplate damage [3], Modic Type I changes [4], and lumbar multifidus atrophy [5] and activation impairment [6,7]. The lumbar multifidus provides compression to the intervertebral segments and plays a critical role in ensuring spinal integrity [8]. Many of the recommended non-pharmacological interventions for non-specific CLBP [9] do not directly target muscle function: some, such as cognitive behavioral therapy and mindfulness-based stress reduction, address its social and psychological aspects, while others (e.g., spinal manipulation) seek to reduce pain. Additionally, while targeted back training demonstrated the ability to both increase the maximum voluntary contraction of the multifidus [10] and improve the multifidus/erector spinae activation ratio [11], volitional exercise is not always feasible for individuals with CLBP [12]. From a public health perspective, alternative interventions that improve multifidus function are desirable, and more rehabilitation options are needed that target strengthening the deep muscles in the back.

Neuromuscular electrical stimulation (NMES) is a form of low-frequency transcutaneous electrotherapy that preferentially stimulates alpha motor neurons, causing involuntary muscular contraction [13]. In therapeutic contexts, clinicians apply NMES prior to volitional exercise to ‘activate’ or improve neural drive to a target muscle [14] with the goal of improving function. A recent RCT found that a single session of NMES, applied prior to motor control exercise, improves multifidus activation greater than sham NMES followed by motor control exercise (*p* < 0.005, ES = 0.70) [15]. Although NMES is less efficient at producing movement than volitional exercise [16], these results show the potential for NMES as an adjunct treatment for CLBP.

Shear-wave elastography (SWE) is a non-invasive ultrasound modality that has been used to quantify change in resting lumbar multifidus stiffness following volitional exercise [17,18]. SWE has also been applied to measure multifidus activity in standing and forward-bent positions [19], as well as in prone with limb-loading tasks [6,20]. Considering that individuals with CLBP present with greater resting multifidus stiffness than healthy controls [20,21], acute changes to this parameter may be clinically relevant. To date, however, SWE has not been used to assess the acute effect of an NMES intervention on lumbar multifidus stiffness. Therefore, the primary aim of this study was to assess the immediate effect of a single treatment with the StimaWELL 120MTRS system, a medium-frequency electrotherapy device, on multifidus muscle stiffness at L4 and L5, in prone resting and in standing, in patients with CLBP. The secondary aim was to examine change in pain intensity from pre- to post-treatment.

## 2. Materials and Methods

### 2.1. Study Design

**Figure 1:**
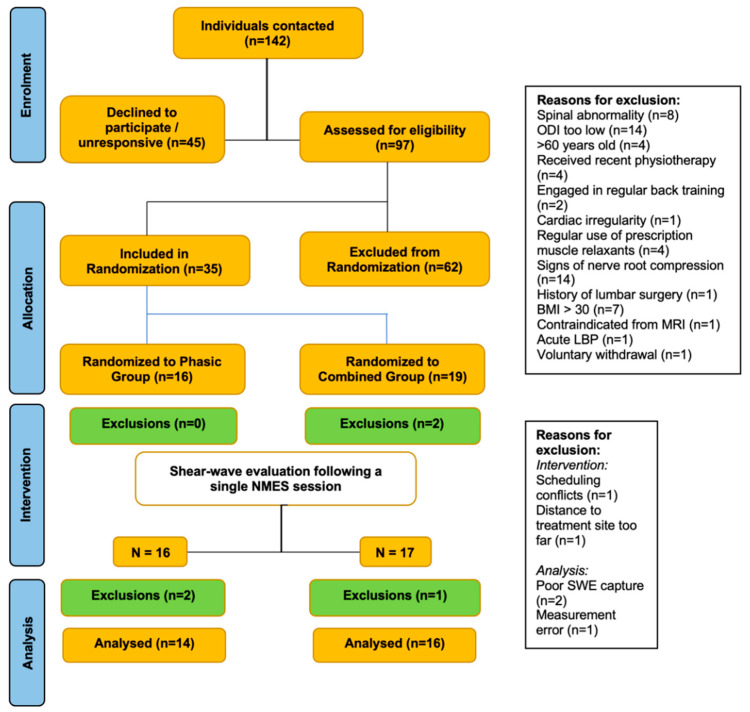
Study flow chart.

Thirty-five participants were randomized into one of two muscle therapy protocols for the lumbar spine using the StimaWELL 120MRTS device (Schwa-Medico, Ehringshausen, Germany): the ‘phasic’ group (3 kHz, modulation 50 Hz) or the ‘combined’ group (3 kHz, modulation 4 Hz and 50 Hz). Participants were recruited from a broader RCT examining the effect of 10 weeks of twice-weekly muscle therapy intervention on CLBP (see study flow diagram, Figure 1). Briefly, the protocols were chosen to target both Type I and Type II muscle fibers, with an emphasis on the latter, which may undergo a shift to Type I fibers in the superficial multifidus in presence of CLBP [6]. The randomization procedures and rationale for the protocols we selected are described in greater detail in the published study protocol paper [22]. For the current study, data were collected during participants’ third NMES treatment. The inclusion and exclusion criteria, listed below, are in line with norms for studies of CLBP involving transcutaneous electrotherapy [14] and ultrasound measurements [15].

Inclusion Criteria

- Chronic non-specific LBP (>3 months), defined as pain in the region between the lower ribs and gluteal folds, with or without leg pain.
- Aged between 18 to 60 years old.
- English or French speakers.
- Have at least score of ‘moderate’ on the Modified Oswestry Disability Index (ODI).
- Able to undergo MRI exam.

Exclusion Criteria

- Currently undergoing or having received physical therapy treatment in the previous month.
- Consistent motor control training for the low back and/or consistent weightlifting, powerlifting, bodybuilding, or strongman training in the previous 6 weeks.
- History of lumbar surgery.
- Presence of positive lumbosacral dermatomes or myotomes.
- Presence of disease which could affect the stiffness of muscle tissue (collagen tissue disease, hemiplegia, multiple sclerosis, or blood clots).
- Presence of systemic disease (cancer, metabolic syndrome).
- Presence of spinal abnormality (spinal stenosis, fracture, infection, tumor, or lumbar scoliosis greater than 10 degrees).
- BMI > 30.
- Presence of cardiac arrhythmia.
- Pregnant and breastfeeding women.
- Individuals with epilepsy.
- Individuals at risk for serious bleeding.
- Individuals with pacemakers or metal implants.
- Individuals with aneurysms or heart valve clips.
- Individuals who have taken prescribed muscle relaxants more than once a week in the previous month.

### 2.2. Study Setting, Ethics Approval, and Intervention

**Figure 2:**
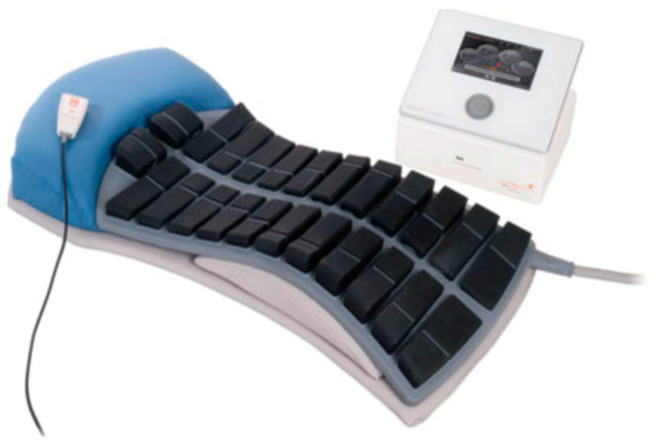
StimaWELL 120MTRS system.

This study was conducted at the School of Health (Concordia University, Montreal, QC, Canada) and received ethics approval from the Central Ethics Research Committee of the Quebec Minister of Health and Social Services (#CCER-20-21-07). All participants provided informed consent prior to the intervention, which was provided with the StimaWELL 120MTRS system (V 3.5), a pre-modulated IFC (interferential current) electrotherapy device that heats up to 40 °C (Figure 2).

### 2.3. Protocol

Upon arrival, participants assessed their current LBP using the numerical pain rating scale (NPRS), a valid assessment tool of LBP intensity [23]. Next, we acquired ultrasound images of the lumbar multifidus at L4–L5 and L5-S1, in prone rest and then in standing, using the shear-wave elastography feature of the Aixplorer ultrasound unit (Supersonic Imagine, Aix-en-Provence, France). For prone imaging, participants lay on their stomach, with a pillow placed under the hips to minimize lumber lordosis (maximum of 10° measured with an inclinometer). We took three images of the multifidus per side, per level. For the standing SWE multifidus measurements, participants stood barefoot on the floor with their arms relaxed. Again, we took three images per side, per level, and care was taken to avoid applied excessive pressure on the skin with the transducer. Measurement procedures are described in full in the protocol paper cited above. The reliability of the measurement is high [ICC = 0.92] for the multifidus at rest at L4–L5 [24].

After baseline imagine, participants received 20 min of treatment with either the ‘phasic’ or ‘combined’ protocol, following which they immediately re-assessed their current level of LBP. They were instructed to gently move or rest, depending on their preference, for approximately 10 min, before lying in prone rest for 5 min. Approximately 15 min post-treatment, we re-measured lumbar multifidus stiffness in prone and standing positions with the same protocol used at baseline.

### 2.4. Statistical Analysis

The mean Young’s modulus (per side, level, and time-point) was calculated and used in the analysis. Because skeletal muscle cannot be assumed to be isotropic, Young’s modulus was divided by three to obtain the shear modulus, which is common practice in musculoskeletal SWE research [20,25]. Statistical analysis was performed with SPSS (IBM SPSS Statistics for Mac, Version 29.0). Normality assumptions were first verified with histogram analysis and Shapiro–Wilk tests, and evaluation of skewness and kurtosis. Baseline demographic characteristics (gender, age, BMI, duration of LBP) were compared, by treatment group, using the Chi-square test and independent *t*-tests. Within-subject changes were analyzed with paired *t*-tests and Wilcoxon sign rank tests, while between-subject differences were assessed with independent *t*-tests and Mann–Whitney U tests. Statistical significance was set at *p* < 0.05.

## 3. Results

Two participants withdrew from the broader RCT prior to the assessments we conducted for this study. Additionally, we chose to exclude data from three participants: two due to poor SWE capture, and one due to measurement error. Images from thirty participants were included in the final analysis: sixteen in the combined group and fourteen in the phasic group. There were no significant differences in baseline characteristics (gender, age, BMI, and duration of LBP) between the two groups, which are reported below in Table 1.

For prone SWE measurements, Wilcoxon sign rank tests revealed significant increases in shear-modulus following a single treatment session in the combined group at right L4 (mean difference = 1.9, 95% CI = 0.8, 2.3, *p* = 0.001), right L5 (MD = 1.2, 95% CI = 0.1, 2.3, *p* = 0.017), and left L5 (MD = 1.0, 95% CI = −0.2, 2.2, *p* = 0.020). There were no significant within-group changes at left L4 in the combined group, or in the phasic group at any level/side. For the between-group comparison, a Mann–Whitney U test revealed that the combined group had a significant greater increase in stiffness at right L4 (*p* < 0.001) post-treatment, but no other significant differences were found. Mean prone multifidus stiffness measurements are reported below in Table 2.

With respect to standing SWE measurements, a paired *t*-test revealed a significant decrease in shear modulus in the combined group at right L4 (MD = −3.8, 95% CI = −6.7, −0.8, *p* = 0.015) and left L5 (MD = −2.1, 95% CI = −4.1, −0.1, *p* = 0.036), and no other significant within-group changes. For the between-group comparison, an independent *t*-test revealed significant differences in shear modulus at left L5 (MD = −3.9, 95% CI = −7.2, −0.7, *p* = 0.016). No other significant differences were found. Mean standing multifidus stiffness measurements are reported below in Table 3.

There were significant decreases in pain intensity in the both groups [(phasic MD = 1.42, 95% CI = 0.68, 2.16, *p* = 0.001); (combined MD = 1.12, 95% CI = 0.34, 1.90, *p* = 0.011)], with no significant between-group differences. Changes in pain intensity are reported below in Table 4.

## 4. Discussion

In this study, we sought to clarify the acute effect of neuromuscular electrical stimulation on multifidus stiffness in CLBP patients. To our knowledge, no studies investigated the immediate effect of NMES or other transcutaneous electrotherapies on multifidus stiffness. Previous research suggests that individuals with CLBP have higher stiffness in prone resting than their asymptomatic counterparts [20,21], with a mean difference at L4-L5 of 1.0 kPA (95% CI = 0.05, 2.0, *p* = 0.04) (adjusted for sex and BMI) [20].

Shear modulus at rest (i.e., when a muscle is minimally contracting) reflects both morphological and physiological properties. Since shear modulus is tissue-dependent [26], elevated multifidus stiffness may reflect changes in muscle composition that have been reported in CLBP populations, such as increased fibrosis or fatty infiltration [27]. Concurrently, shear modulus also reflects muscle function: when a muscle contracts it stiffens, which is positive in the context of force production. Notably, individuals with LBP were reported to have a relatively smaller increase in stiffness of the superficial multifidus (SM) during contraction than healthy controls (1.54 ± 0.47 vs. 2.65 ± 1.36, *p* < 0.003) [6], which may indicate reduced function of this muscle.

True morphological adaptations, such as change in the intramuscular fat-muscle ratio, would not be expected to change following a single training session. However, both increases and decreases in muscle stiffness have been reported following an acute bout of training. Leung et al. (2017) reported significant increases in the stiffness of the medial gastrocnemius (75 ± 47.7%, *p* < 0.001) and lateral gastrocnemius (71 ± 51.8%, *p* < 0.001) in healthy young adults following a single session of 10 × 15 eccentric heel drops [28]. Kumamoto et al. (2020) found that two groups of healthy adults that completed the Biering–Sorenson lumbar endurance test for 1 min had greater shear modulus of the lumbar multifidus at L4-5, post-test, compared to one group that did not complete the test (19.3 ± 9.1 vs. 5. ± 2.9, *p* = 0.005; 19.6 ± 9.1 vs. 5.7 ± 2.9, and *p* = 0.005) [17]. Conversely, Vatovec et al. (2022) reported that an isometric trunk extension protocol, designed to induce fatigue of the paraspinal muscles, resulted in a decrease of −1.22 kPa (*p* = 0.022) and −1.18 kPa (*p* = 0.006) of the superficial and deep multifidus, respectively, in healthy young adults [18]. Chalchat et al. (2020) examined the effect of voluntary isometric contractions of the vastus lateralis (VL) in young men, and reported that a 6 × 10 × 5 s maximal contraction fatiguing protocol resulted in significant decreases in VL stiffness immediately post-intervention (*p* < 0.05), but not 20 min post-intervention [29]. A possible explanation for these differences in muscle stiffness lies in the training protocol used. A protocol designed to induce fatigue, which was the case for Chalchat et al. and Vatovec et al. [18,29], might have reduced neural drive to the target muscles such that resting muscle tone was lower compared to baseline. In such a situation, the CNS would have to produce action potentials at a higher frequency to produce the same level of force as at baseline [30]. On the other hand, warm-ups—which are non-fatiguing, potentiating bouts of training—can increase bat swinging speed [31] and jump power [32]. The increase in power in particular may be driven by increases in nerve conduction velocity, resulting in a higher number of sarcomeres contracting simultaneously [32], then resulting in transient increases in muscle stiffness.

In this study, we observed a significant increase in lumbar multifidus stiffness (at rest in prone) in the combined group following a 20 min muscle therapy intervention with the StimaWELL 120MTRS system in three out of four measurement sites (right L4, *p* = 0.001; right L5, *p* = 0.017; and left L5, *p* = 0.020), but no significant changes in the phasic group. Additionally, we noted a greater uniformity in the combined group’s response to the treatment, which led to mean increases in stiffness at all four measurement sites, whereas the phasic group had mean increases in stiffness at L5 only. There are a number of possible explanations for these observed differences. Firstly, the combined protocol is designed to stimulate both phasic and tonic muscle fibers, and includes an additional sequence compared to the phasic protocol (two sequences versus three sequences). It is possible that the addition of this third sequence had a significant effect on lumbar multifidus activation, specifically of Type I fibers. Secondly, when taking our SWE measurements, we positioned the Q-box just superficial to the zygapophyseal joints. Although we did not intentionally divide our area of measurement into two distinct regions of interest (ROIs), the Q-box position most likely captured the shear modulus of the deep multifidus (DM) to a greater extent than the superficial multifidus (SM) [6]. The phasic protocol primarily targets Type II fibers, whereas the DM has a higher concentration of Type I fibers [33]. As a result, the effect of phasic stimulation on shear modulus may have been underestimated, as it likely influenced the SM, which was not the focus of imaging. On the other hand, the increases in stiffness we identified in the combined group likely does reveal the true effect of that protocol on the DM. Future studies should investigate the SM and DM separately, especially in contexts where these muscles may be preferentially stimulated.

The results of this “single session” intervention suggest mostly non-significant changes in multifidus stiffness in standing, though there was a significant decrease in shear modulus at right L4 (*p* = 0.015) and left L5 (*p* = 0.036) in the combined group. However, when comparing the changes in both groups, we noted a decrease in shear modulus in the combined group at all four measurement sites, whereas the effect in the phasic group was less consistent. This is a surprising finding, considering that shear modulus was increased in prone. However, care must be taken when comparing passive and active muscle stiffness. Humans use a number of different muscle activation strategies depending on the task at hand [34], energy cost, neuromuscular composition of muscles and/or motor units [35], and previous recruitment strategies [36]. In their analysis of load-sharing strategies used during an isometric trunk extension task, Tier et al. (2022), noted a high level of variation in shear modulus of the lumbar multifidus (and EMG), both between individuals and between trials and positions [37]. In particular, they concluded that group data may poorly reflect individual motor recruitment strategies [37]. We observed a similar finding across our standing SWE measurements, with wide standard deviations likely reflecting differences in both load sharing strategies in standing and responses to this NMES therapy session. Additionally, the variation could reflect inter group differences in ‘control’ and ‘movement’ impairments, following O’Sullivan’s classification [38]. If the combined group had a greater relative number of individuals with ‘control’ impairments, where the multifidus is excessively activated, a decrease in standing stiffness in that group could reflect a positive short-term response to the intervention.

With respect to the observed reduction in shear modulus in the combined group, one explanation is that this is the result of an altered standing posture following treatment. The shear modulus of the lumbar multifidus was previously reported to be higher with trunk flexion compared to prone in healthy adults [19,21]. The lumbar multifidus eccentrically contracts with forward trunk bending and posterior pelvic tilt (i.e., ‘stretch’), which accounts for the increases in shear modulus observed in these studies. Interestingly, Creze et al., (2019) did not find significant differences in shear modulus between prone rest and standing trunk extension for either the multifidus or the erector spinae [19], despite the fact that these muscles contract concentrically with active lumbar extension [39]. This suggests that muscle length may play a relatively greater role than muscle tone in determining shear modulus. Due to the design of the StimaWELL120MTRS system, which is comprised of bilateral stimulation pads across multiple spinal levels, its muscle stimulation protocols stimulate the lumbar spine globally rather than muscles in isolation, unlike traditional NMES. It is possible that the combined protocol increased overall muscle tone of the paraspinal muscles such as to induce a change in posture towards one of relatively greater lumbar extension; if so, the net effect might be to lower multifidus stiffness in standing, compared to pre-treatment, due to muscle shortening. However, this rationale should be considered with caution, since we did not directly assess change in standing lumbar lordosis from pre- to post-treatment. Additionally, it is likely that there was significant variability in the postural strategies that our participants adopted, making it difficult to draw definite conclusions about the effect of the single therapy session on standing stiffness.

The results of our study suggest that treatment with the combined setting likely had a potentiating effect, via increased muscle tone, of the multifidus in prone. Although NMES places a greater metabolic demand on muscle than volitional contractions [40] and induces a higher rate of muscle fatigue [41], the muscle stimulation protocols used in this study differ from classical NMES in a number of ways. Firstly, all StimaWELL 120MTRS system protocols stimulate spinal regions (lumbar, thoracic, etc.) rather than isolated muscles, such that metabolic demand was likely shared by both the multifidus and erector spinae across multiple spinal levels. Secondly, the protocols used in this study cycle through 2–3 stimulation sequences, with current flowing through individual channels at variable durations and intensities. Third, the stimulation frequencies we used did not exceed 50 Hz, which is on the lower end of what is considered necessary to produce maximal tetanic force [40]. Considered together, it is unlikely that these protocols maximally fatigued the lumbar multifidus, allowing for a priming effect in the combined group (as reflected by the observed increases in prone shear modulus).

### 4.1. Elevated Resting Muscle Stiffness–Is It a Good Thing?

The relationship between resting muscle stiffness and function appears to be both tissue- and position-dependent. In contexts where muscle will need to be moved through a full range of motion, interventions that acutely reduce muscle stiffness are likely beneficial. For instance, Iwata et al. (2019) reported that an active stretching protocol for the hamstrings (10 × 15 × 30 s) in healthy volunteers increased hamstring ROM and decreased stiffness (measured in Nm/°) compared to both controls and pre-intervention values [42]. On the other hand, some stiffness of tonic muscles is necessary for maintaining posture. An inter-muscular network analysis of synchronous muscle fiber activation in healthy young adults revealed ‘physiologically significant synchronization of muscle fibers only within the BackL-BackR sub-network’ in supine rest [34], suggesting that even at rest, an elevated level of paraspinal muscle tone relative to other muscles is a normative finding. Importantly, this relatively high level of resting paraspinal muscle tone suggests significant Type I fibers activity. Given that lumbar multifidus function is impaired in CLBP patients [6], transient increases in resting multifidus stiffness following a muscle stimulation intervention, reflecting an acute elevation of muscle tone, might translate to improved short-term function of the multifidus as a spine-stabilizer. Of note, higher SWE values may be independent of self-perceived stiffness, as reported by Dietrich et al. (2020) in their study of women with chronic neck pain [43].

Finally, a single muscle therapy treatment with the StimaWELL 120MTRS system led to statistically significant reductions in pain in both treatment groups, despite variable SWE responses to the intervention. To our knowledge, this is the first report on the acute effect of a single electromyostimulation (EMS) session on low back pain. Although previous research found EMS to be effective at reducing pain intensity in CLBP patients [44,45,46], those studies involved multiple treatment sessions over the course of several weeks. Our results are even more notable given that pain reduction is not a primary aim of NMES-type treatments [13]. Though our results do not reach the threshold for minimal clinically important difference (MCID) in pain intensity, which is 2 points on an 11-point NPRS [23], they point to benefits of muscle therapy treatment with the StimaWELL 120MTRS that extend beyond its direct effect on the paraspinal musculature.

### 4.2. Limitations

This study suffered from a number of methodological limitations. Firstly, as we lacked a true control group, we did not examine change in multifidus stiffness in individuals who did not undergo treatment. Although sham electrotherapy is sometimes used as a control in studies that measure the subjective effects of electrotherapy (in which the minimum therapeutic dose is provided [47]), we considered it plausible that this type of control could affect multifidus stiffness, thereby neutering its effect. Second, assessments were not uniformly conducted during participants’ third visit, due to scheduling conflicts and equipment malfunction. Overall, the acute change in multifidus stiffness was measured during the third visit in twenty-three participants, during the fourth visit in four participants, fifth in two, and sixth in one. However, this variability is unlikely to have affected our results, as all assessments were carried out within the first three weeks of the intervention, which is prior to onset of any potential hypertrophic [40]. Third, due to the design of the StimaWELL 120MTRS system and of its preset programs, we were unable to target discrete segments of the lumbar multifidus muscle; rather, electrical current was applied to the paraspinal musculature across its length and width. Whereas traditional NMES involves the application of an adhesive pad to a target region that is stimulated with electrical current [13], with observable muscle contraction when the area is visible, treatment with the StimaWELL 120MTRS system occurs with participants lying supine, obscuring the precise site of stimulation at a given moment. While the current was increased to tolerance for all participants, who were told that they should be experiencing contractions or pulsations in their lumbar spine, without visibility of the multifidus, we cannot guarantee that the intensity of stimulation was high enough to evoke muscle contractions at all times. However, the design of the intervention precludes our making a direct comparison in terms of efficacy with other forms of NMES. Lastly, we did not examine other biomechanical or functional outcomes, such as isokinetic paraspinal muscle strength, lumbar range of motion, or standing posture, and cannot speak to possible correlations between these variables and change in lumbar multifidus stiffness.

## 5. Conclusions

In sum, 20 min of treatment with two of the StimaWELL 120MTRS system’s muscle stimulation programs resulted in significant increases in resting multifidus stiffness in the combined, but not the phasic, group. There were mild within-group changes in multifidus stiffness in standing; significant decreases at right L4 and left L5 in the combined group were found, but no other significant changes. Overall, results are more consistent in the combined than in the phasic group, both in prone and standing. The combined program for lumbar muscle therapy appears to acutely increase prone multifidus stiffness in CLBP patients, but both groups experienced significant reductions in low back pain. Research into the effect of pin-point EMS interventions (i.e., right lumbar multifidus from L3 to 5) on paraspinal muscle stiffness is warranted.

## Figures and Tables

**Table 1:** Baseline characteristics (mean + SD).

	Combined Group (*n* = 16)	Phasic Group (*n* = 14)	Significance
Sex	7 male; 9 female	6 male; 8 female	0.961 ^&^
Age (yrs)	42.2 ± 12.5	42.6 ± 12	0.760 ^
BMI	24.7 ± 2.3	24.2 ± 3	0.609 ^
Duration of LBP (months)	113.5 ± 112.9	77.3 ± 80.6	0.327 ^

^&^ denotes Pearson Chi-square test; ^ denotes independent *t*-test.

**Table 2:** Mean prone multifidus stiffness in kPA [± = SD, {95% CI}].

	Combined Group (*n* = 16)	Phasic Group (*n* = 14)	Between-Group Difference (Combined Minus Phasic)
L4 level			
Right Pre	4.1 ± 1.4	5.3 ± 2.2	NA, *p* < 0.001 ^#^
Right Post	6.0 ± 2.3	4.8 ± 1.9	
Difference (post-pre)	1.9 [0.8, 3.0], *p* = 0.001 ^$^	−0.4 [−1.3, 0.3], *p* = 0.224 ^a^	
Left Pre	4.5 ± 1.8	6.5 ± 2.6	0.63 {−1.2, 2.4}, *p* = 0.493 ^^^
Left Post	4.9 ± 1.8	6.4 ± 2.8	
Difference (post-pre)	0.4 [−0.6, 1.5] *p* = 0.304 ^a^	−0.1 [−1.7, 1.5] *p* = 0.908 ^a^	
L5 level			
Right Pre	4.2 ± 1.2	4.8 ± 2.4	NA, *p* = 0.525 ^#^
Right Post	5.3 ± 2.6	5.2 ± 1.9	
Difference (post-pre)	1.2 [0.1, 2.3] *p* = 0.017 ^$^	0.45 [−0.3, 1.2] *p* = 0.226 ^a^	
Left Pre	4.2 ± 2.1	4.9 ± 2.7	NA, *p* = 0.608 ^#^
Left Post	5.2 ± 2.6	5.3 ± 2.8	
Difference (post-pre)	1.0 [−0.2, 2.2], *p* = 0.020 ^$^	0.4 [−0.5, 1.5] *p* = 0.354 ^a^	

^a^ denotes paired *t*-test; ^ denotes independent *t*-test; ^$^ denotes Wilcoxon sign rank test; and ^#^ denotes Mann–Whitney U test. **Bold** denotes statistical significance (*p* < 0.05).

**Table 3:** Mean standing multifidus stiffness in kPA [± = SD, {95% CI}].

	Combined Group (*n* = 16)	Phasic Group (*n* = 14)	Between-Group Difference (Combined Minus Phasic)
L4 level			
Right Pre	18.2 ± 7.7	12.5 ± 8.5	−1.0 [−5.3, 3.1], *p* = 0.596 ^^^
Right Post	14.3 ± 7.2	9.8 ± 4.7	
Difference (post-pre)	−3.8 [−6.7, −0.8], *p* = 0.015 ^a^	−2.7 [−5.9, 0.5], *p* = 0.097 ^a^	
Left Pre	13.5 ± 7.3	11.7 ± 8.7	NA, *p* = 0.812 ^#^
Left Post	13.2 ± 6.6	10.8 ± 7.3	
Difference (post-pre)	−0.3 [−2.6, 2.0] *p* = 0.778 ^a^	−0.9 [−2.9, 1.1] *p* = 0.345 ^$^	
L5 level			
Right Pre	19.3 ± 11.4	14.6 ± 9.3	−2.6 [−7.2, 1.9], *p* = 0.255 ^^^
Right Post	17.5 ± 12.1	15.8 ± 9.2	
Difference (post-pre)	−1.8 [−5.4, 1.7] *p* = 0.294 ^a^	0.7 [−2.3, 3.9] *p* = 0.426 ^a^	
Left Pre	16.4 ± 10.1	14.7 ± 9.8	−3.9 [−7.2, −0.7], *p* = 0.016 ^^^
Left Post	14.3 ± 9.2	16.5 ± 12.0	
Difference (post-pre)	−2.1 [−4.1, −0.1], *p* = 0.036 ^a^	1.8 [−0.8, 4.4] *p* = 0.165 ^a^	

^a^ denotes paired *t*-test; ^ denotes independent *t*-test; ^$^ denotes Wilcoxon sign rank test; and ^#^ denotes Mann–Whitney U test. **Bold** denotes statistical significance (*p* < 0.05).

**Table 4:** Change in pain intensity [± = SD, {95% CI}].

	Combined Group (*n* = 16)	Phasic Group (*n* = 14)	Between-Group Difference (Combined Minus Phasic)
Pain Pre-Treatment	4.18 ± 1.72	4.57 ± 1.69	NA, *p* = 0.637 ^#^
Pain Post-Treatment	3.06 ± 1.91	3.14 ± 1.79	
Difference (pre-post)	1.12 [0.34, 1.90] ***p* = 0.011** ^$^	1.42 [0.68, 2.16] ***p* = 0.001** ^a^	

^a^ denotes paired *t*-test; ^$^ denotes Wilcoxon sign rank test; and ^#^ denotes Mann–Whitney U test. **Bold** denotes statistical significance (*p* < 0.05).

## Data Availability

The original contributions presented in the study are included in the article, further inquiries can be directed to the corresponding author.
